# Alpha-Synuclein Post-translational Modifications: Implications for Pathogenesis of Lewy Body Disorders

**DOI:** 10.3389/fnagi.2021.690293

**Published:** 2021-06-25

**Authors:** Nelson de Oliveira Manzanza, Lucia Sedlackova, Raj N. Kalaria

**Affiliations:** ^1^Translational and Clinical Research Institute, Campus for Ageing and Vitality, Newcastle University, Newcastle upon Tyne, United Kingdom; ^2^Biosciences Institute, Campus for Ageing and Vitality, Newcastle University, Newcastle upon Tyne, United Kingdom

**Keywords:** alpha synuclein, neuronal membrane, Lewy body disorders, post-translational modifications, dementia

## Abstract

Lewy Body Disorders (LBDs) lie within the spectrum of age-related neurodegenerative diseases now frequently categorized as the synucleinopathies. LBDs are considered to be among the second most common form of neurodegenerative dementias after Alzheimer's disease. They are progressive conditions with variable clinical symptoms embodied within specific cognitive and behavioral disorders. There are currently no effective treatments for LBDs. LBDs are histopathologically characterized by the presence of abnormal neuronal inclusions commonly known as Lewy Bodies (LBs) and extracellular Lewy Neurites (LNs). The inclusions predominantly comprise aggregates of alpha-synuclein (aSyn). It has been proposed that post-translational modifications (PTMs) such as aSyn phosphorylation, ubiquitination SUMOylation, Nitration, o-GlcNacylation, and Truncation play important roles in the formation of toxic forms of the protein, which consequently facilitates the formation of these inclusions. This review focuses on the role of different PTMs in aSyn in the pathogenesis of LBDs. We highlight how these PTMs interact with aSyn to promote misfolding and aggregation and interplay with cell membranes leading to the potential functional and pathogenic consequences detected so far, and their involvement in the development of LBDs.

## Highlights

- aSyn aggregation and transformation into LBs and LNs underlie progression of LBDs and other synucleinopathies.
- The failure of proteostasis network is linked to maintain biologically active aSyn.
- Post-translational modifications (PTMs) contribute to aSyn pathogenicity.
- aSyn PTMs include ubiquitination, nitration, acetylation, SUMOylation, glutathionylation, Glycosylation, and phosphorylation.
- PTMs interventions or modifications may reduce accumulation of LBs and prevent LBDs.

## Introduction

**Figure 1:**
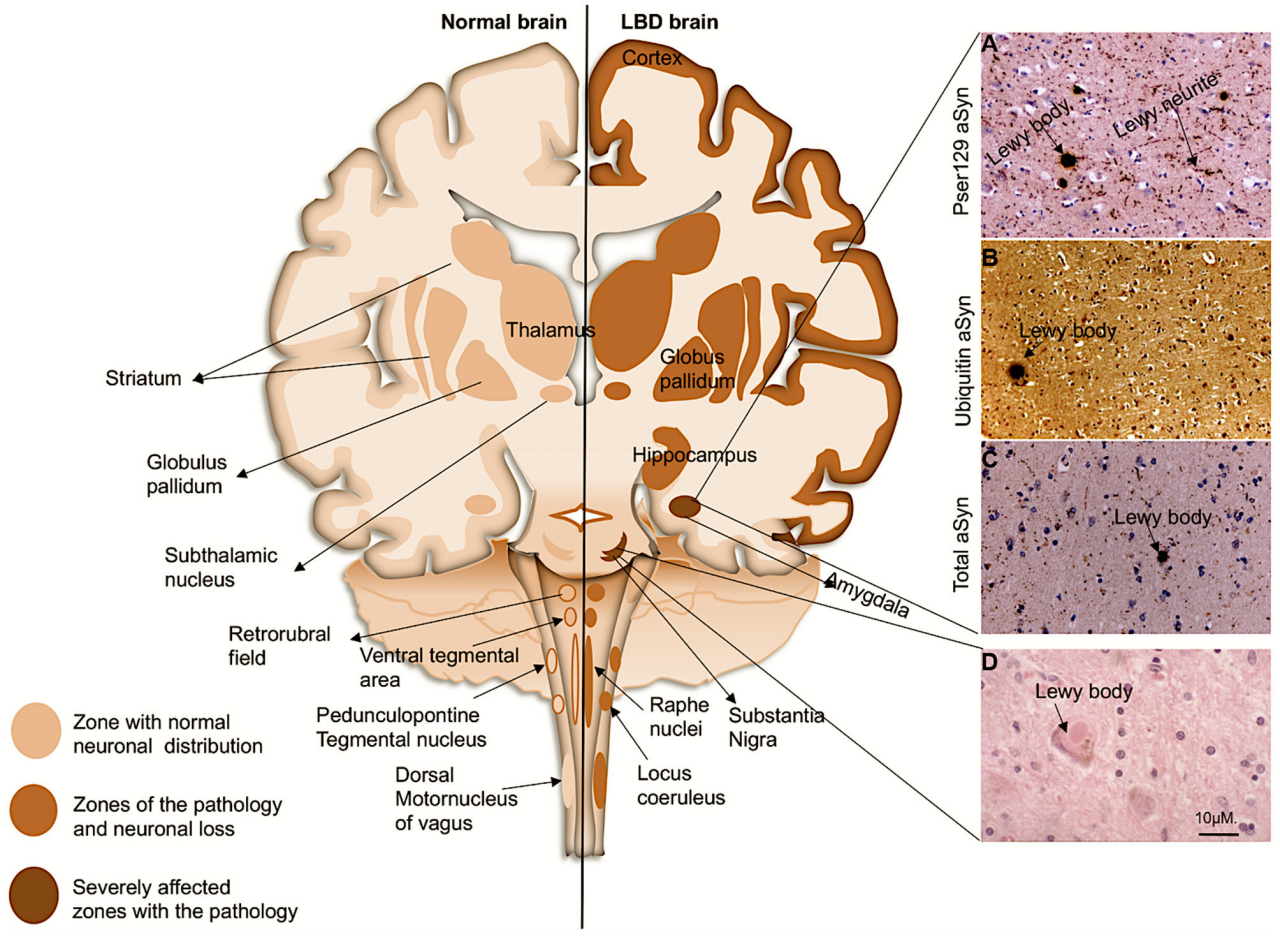
Coronal schematic of the brain, depicting normal structures and in LBD subjects. Shading shows the distribution of aSyn in LBs/LNs in cortical and subcortical regions. Subcortical regions are associated with PD, and as the disease progresses to cortical regions it corresponds with DLB and PDD. (Right, **A–D**): LB pathology (arrows) revealed in the amygdala of a patient with DLB with antibody to pSer^129^ aSyn **(A)**, ubiquitin **(B)**, and whole aSyn counterstained with haematoxylin. **(D)**, LB with a halo typically found in the substantia nigra. Magnification bar: 10μM.

Lewy body dementias (LBDs) are progressive neurodegenerative disorders characterized by the presence of abnormal intra-neuronal deposits commonly called Lewy bodies (LBs) and deposition of extracellular Lewy neurites (LNs) (Figure 1) (McKeith et al., 2017; Outeiro et al., 2019). LBDs are currently regarded to encompass dementia with Lewy Bodies (DLB), Parkinson's disease (PD), and Parkinson's disease with dementia (PDD). They are the second most prevalent types of neurodegenerative dementia, after Alzheimer's disease (AD), accounting for ~22% of all dementias in older people. The incidence of LBDs is associated with advancing age as it increasingly manifests in individuals over 65 years of age (Spillantini et al., 1998; Braak et al., 1999; McKeith et al., 2003, 2004, 2017; Yang et al., 2018; Outeiro et al., 2019). The clinical and pathological profiles of the LBDs demonstrate considerable overlap among the subtypes. Patients diagnosed with DLB often present with parkinsonism associated with brainstem and midbrain pathology and many PD cases present with dementia owing to pathological involvement of the limbic and cortical structures progressing to PDD (Figure 1) (Jellinger and Korczyn, 2018). The differential diagnosis between PDD and DLB is achieved using the temporal profile during onset of disease such as the appearance of cognitive symptoms after the presentation of Parkinsonian features. Thus, if the onset of dementia occurs at least 1 year after the onset of initial PD related symptoms, the diagnosis is consistent with PDD. In contrast, if the onset of dementia begins within 1 year of the development of parkinsonism, then the profile is consistent with DLB (Spillantini et al., 1997, 1998; Gurd et al., 2000; McKeith, 2006; Goedert et al., 2013; McKeith et al., 2017). If dementia develops in the context of established PD, then it is consistent with PDD (McKeith et al., 2017). Generally, PDD develops after ~10 years of the onset of PD (Aarsland et al., 2003; McKeith et al., 2017). However, the neuropsychological profile of PDD is not phenotypically very different compared with DLB (Aarsland et al., 2003). Despite progress in our understanding and identification of the variable symptoms, which may be indicative of either PD or DLB disease progression, a definitive clinical diagnosis remains difficult to achieve. At present, a confirmed diagnosis is only provided through post-mortem examination by the detection of intraneuronal inclusions, consistent with LB pathology found in the neuronal soma and LN pathology within the neuronal processes.

Upon light microscopy, LBs are characteristically seen as neuronal inclusions usually eosinophilic round bodies surrounded by a halo (Figure 1). LBs form in subsets of large or long projection neurons which seem to be prone to such accumulation particularly monoaminergic neurons in the brainstem and midbrain (Halliday et al., 1990; Braak et al., 2004). However, in the limbic system and the neocortical areas, LBs are most frequently found in glutamatergic pyramidal neurons; LBs have also been reported in cholinergic neurons of the basal forebrain (Wakabayashi et al., 1995; Marui et al., 2003; Dugger and Dickson, 2010; Bernstein et al., 2011). LBs and LNs are not exclusive to LBDs, as they also occur in other neurodegenerative diseases each defined by different regional and cellular distributions and collectively termed as the synucleinopathies (Spillantini et al., 1997, 1998; Mattila et al., 2000; Goedert et al., 2013).

LBs and LNs were originally immunohistochemically identified by antibodies to ubiquitin. However, it is now standard to demonstrate them with antibodies to alpha-synuclein (aSyn) (Yoshimoto et al., 1995; Spillantini et al., 1998). aSyn protein is the most abundant constituent of the inclusions (Spillantini et al., 1997, 1998; Baba et al., 1998). More recent studies have shown that LBs and LNs also comprise many cellular components including dysmorphic organelles, vesicular structures and membranes (Spillantini et al., 1997, 1998; Braak et al., 1999; Shults, 2006; Shahmoradian et al., 2018). aSyn protein was first identified in 1988, as a small intracellular protein, and it was named so because of its localization in the nuclear envelopes and synaptic vesicles of the cells (Maroteaux et al., 1988). aSyn belongs to a family of three homologous synuclein proteins, which include β-synuclein and γ-synuclein (Nakajo et al., 1990; Jakes et al., 1994; Buchman et al., 1998; Lavedan et al., 1998; Wales et al., 2013). Subsequently, Saitoh and colleagues discovered that aSyn was associated with amyloid plaques of Alzheimer's disease (AD) patients (Kalaria, 1997) and called it the non-amyloid beta component (NAC) (Ueda et al., 1993). aSyn further became a major focus in LBDs upon discovery of point mutations in the *aSyn* gene in familial forms of PD (Polymeropoulos et al., 1997; Nemani et al., 2010). Point mutations within the aSyn encoding gene (*SNCA)* leading to formation of LBs and LNs has largely confirmed its pathogenic role in LBDs (Polymeropoulos et al., 1997; Bussell and Eliezer, 2001; Kruger et al., 2001; Zarranz et al., 2004; Lesage et al., 2013; Proukakis et al., 2013; Pasanen et al., 2014).

**Figure 2:**
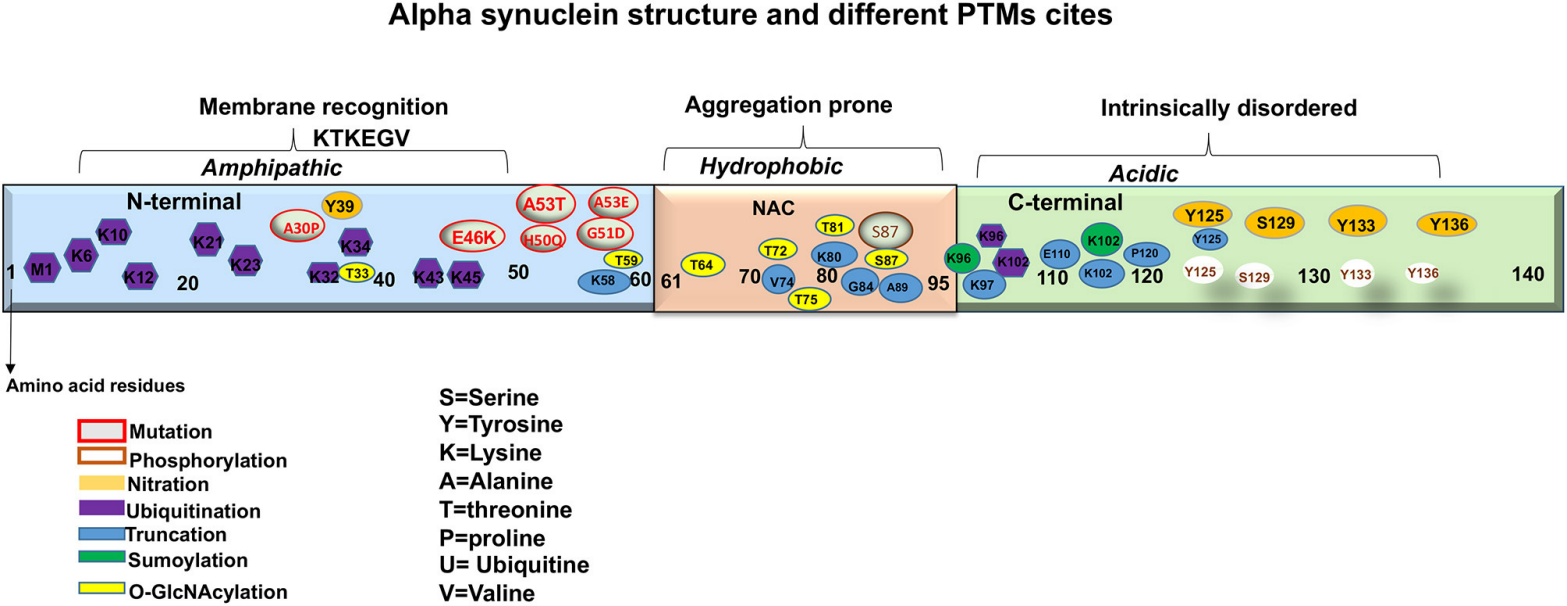
A representation of aSyn structure and main sites of mutations and post-translational modifications (PTMs).

Although the exact mechanism that triggers the aggregation of aSyn and the consequent inclusion formation is not clearly understood it is reported that the influence of mutations and post-translational modifications (PTMs) add to the protein pathogenicity. It is thought that the PTMs play important roles in impairing the native state of the protein, which consequently induces protein misfolding and aggregation (Villar-Pique et al., 2016). Thus, it is important to assess the disease associated PTMs of aSyn, as it may enable elucidation of potential processes involved in its pathological aggregation (Jellinger and Korczyn, 2018; Moors et al., 2018). In addition, it may help to elucidate the observed anatomical and distribution patterns of LBs seen within the LBDs, as well as provide better insights in strategies for treatments. This review outlines the involvement of different PTMs of aSyn described in LBDs, with particular focus on aSyn phosphorylation, ubiquitination SUMOylation, Nitration, o-GlcNacylation, and Truncation (Figure 2). Our aim is to provide insight into how the PTMs affect aSyn to misfold and aggregate, leading to the potential functional and pathogenic consequences so far detected, that lead to the development of LBDs.

## aSyn Structure

aSyn is ubiquitously expressed in the CNS comprising ~1% of total cytosolic neuronal protein (Kahle, 2008). It is a relatively small protein with molecular weight of 14.5 kDa, nearly 4 times as large as the amyloid-β peptide. Synucleins are predominantly neuronal proteins, most abundant in presynaptic terminals (Maroteaux et al., 1988; Ueda et al., 1993). aSyn is a product of the synuclein alpha (*SNCA)* gene, located at position 21 of the long arm of chromosome 4. The gene encodes 140 highly charged amino acid (aa) residues, which do not adopt a defined structure in an aqueous solution (Polymeropoulos et al., 1997; Wales et al., 2013). aSyn consists of three distinct domains: *The N-terminal, the Central* and *C terminal domains* (Figure 2). The *N-terminal domain or the amino terminus*, (aa 1-60) contains four 11-dimer repeats that contain a KTKEGV consensus sequence. This region is predisposed to fold into alpha helices and has been hypothesized to be linked with the lipid binding capacity of the protein (Polymeropoulos et al., 1997; Marui et al., 2004; Wales et al., 2013). The *Central domain*, also called the *NAC* (aa 61-95) is particularly hydrophobic and amyloidogenic (Maroteaux et al., 1988; Ueda et al., 1993). Studies have reported that this domain is associated with aSyn aggregation when it adopts a beta-sheet structure. Moreover, identified mutations that are linked with synucleinopathies are also found in this region (Figure 2) (Polymeropoulos et al., 1997; Lesage et al., 2013; Proukakis et al., 2013). Finally, the *C terminal domain* or *the carboxyl terminus* (aa 96-140), provides flexibility to the polypeptide, because of its abundance in proline residues, that are known to affect the secondary structure of the region (Maroteaux et al., 1988; Ueda et al., 1993). In addition, this region is highly acidic, intrinsically unstructured and is thought to be the target of diverse PTMs (Figure 2) (Davidson et al., 1998; Bussell and Eliezer, 2001, 2003). Furthermore, this region is also involved in protein-protein interactions, polyamine binding, protection from protein aggregation and modulation of membrane binding properties of aSyn (Crowther et al., 1998; Nielsen et al., 2001; Park et al., 2002; Fernandez et al., 2004; Brown, 2007; Bodner et al., 2009; Oueslati et al., 2010; Sevcsik et al., 2011).

## aSyn Function

The precise function of aSyn remains the focus of intense debate and research. The predominant cytoplasmic expression of aSyn and its specific transport to presynaptic terminals, suggests a regulatory function within the synapse (Burre, 2015). Modulation of aSyn expression by either overexpression, knock-down or knockout, alters synaptic vesicle trafficking, and maintenance, as well as neurotransmitter compartmentalisation, storage, release, recycling, and changes in synaptic activity and plasticity (Lashuel et al., 2012; Burre et al., 2013; Allen Reish and Standaert, 2015). Given the extensive nature of the synaptic parameters affected, it is plausible that aSyn may act as an accessory/adaptor protein for these processes. aSyn is thought to empower a number of better-defined synaptic proteins. aSyn supports N-ethylmaleimide-sensitive factor attachment protein receptor (SNARE) proteins in their role, potentially acting with some level of functional redundancy with Cysteine Stirring Protein alpha (CSP-α) 1- (Goda, 1997; Gerst, 1999; Burre et al., 2010; Lautenschlager et al., 2017; Lou et al., 2017; Hawk et al., 2019; Agliardi et al., 2021). aSyn is thought to assist in the formation of SNARE complexes via the direct binding of synaptobrevin 2. Such facilitation of SNARE complex formation ultimately influences the dynamics of synaptic vesicle cycling during neurotransmitter release (Table 1) (Burre et al., 2010). An interesting study by Fortin et al. (2005), supports this, as it demonstrated that during neural activity, fluorescently labeled aSyn retracts from synaptic vesicles and then returns progressively to the vesicles (Fortin et al., 2005). Nevertheless, despite these research efforts the full extent of aSyn function(s) remains poorly understood.

**Table 1:** Summary of aSyn function, targets, action, and effects.

**aSyn Function**	**Target**	**Action**	**Effects**	**References**
Chaperone activity	Presynaptic membrane Heat shock proteins	Maintenance of SNARE complex Helping protein to obtain correct conformations	SNARE complex assembly Efficient neurotransmitter release	Burre et al., 2010; Burré et al., 2014; Choi et al., 2013
Antioxidation	Cytochrome c oxidase JNK-interacting protein	Inhibition of Caspases activation Inhibition of JNK pathway	Neuroprotection	Hashimoto et al., 2002; Zhu et al., 2006
Maintenance of PUFA Levels	Acyl-coA synthetase	Regulation of lipid synthesis	Synthesis of brain vital fatty acids	Ruipérez et al., 2010
Neuronal differentiation	Rab3a	ERK/MAPK pathway activation	Gene transcription	Ostrerova et al., 1999; Chen et al., 2013
Regulation of Calmodulin activity	CaM	Activation of CaM	Modulation of G-protein-coupled receptor kinase	Martinez et al., 2003
Regulation of glucose levels	G-protein-coupled receptor Pancreatic β-cell KATP channel	Insulin inhibition	Susceptibility to diabetes	Geng et al., 2011; Rodriguez-Araujo et al., 2015; Sharma et al., 2015
Regulation of DA biosynthesis	Protein phosphatase 2A	Inhibition of tyrosine Hydroxylase	Regulation of DA levels	Peng et al., 2005; Yang et al., 2013
Suppression of apoptosis	Protein C Kinase	Deactivation of NFkB	Neuroprotection	Jin et al., 2011

*SNARE, sensitive factor attachment protein receptor; PUFA, polyunsaturated fatty acid; MAPK, Mitogen-activated protein kinase; JNK, cJun N-terminal kinase; NFkB, nuclear factor kB; CaM, Calmodulin; DA, dopamine*.

## aSyn Aggregation and Its Role in Cellular Dysfunction

aSyn aggregates are also the main constituents of LBs and LNs. Aggregates are also present in mixed neuropathological cases where both LB pathology and atypical AD with amyloid-β and Tau pathology are present (Spillantini et al., 1997, 1998). Multiple lines of evidence shows that unstructured soluble monomers of aSyn are transformed into oligomers, which then aggregate to form mature insoluble fibrils and form LBs (Harper et al., 1997; Walsh et al., 1997; Lambert et al., 1998; Miake et al., 2002). *In vitro* studies demonstrated that the aggregation process occurs in three different stages, named the *lag, elongation* and *stationary* phases (Buell et al., 2014). In the *lag phase*, the aggregation occurs through key structural changes within the protein resulting in the formation of misfolded monomers and aggregation, culminating in the formation of oligomers. In the *elongation phase*, an exponential growth of oligomers takes place, to form non-soluble fibrils achieved by the continual addition of monomers. In the *stationary phase*, the consumption of monomeric aggregates culminates into reduction of the fibril growth rate and formation of LBs (Invernizzi et al., 2012; Buell et al., 2014). However, this sequence of events appears to be much more complex *in vivo* as different oligomeric forms may be involved in the process (Fairfoul et al., 2016; Shahnawaz et al., 2017).

**Figure 3:**
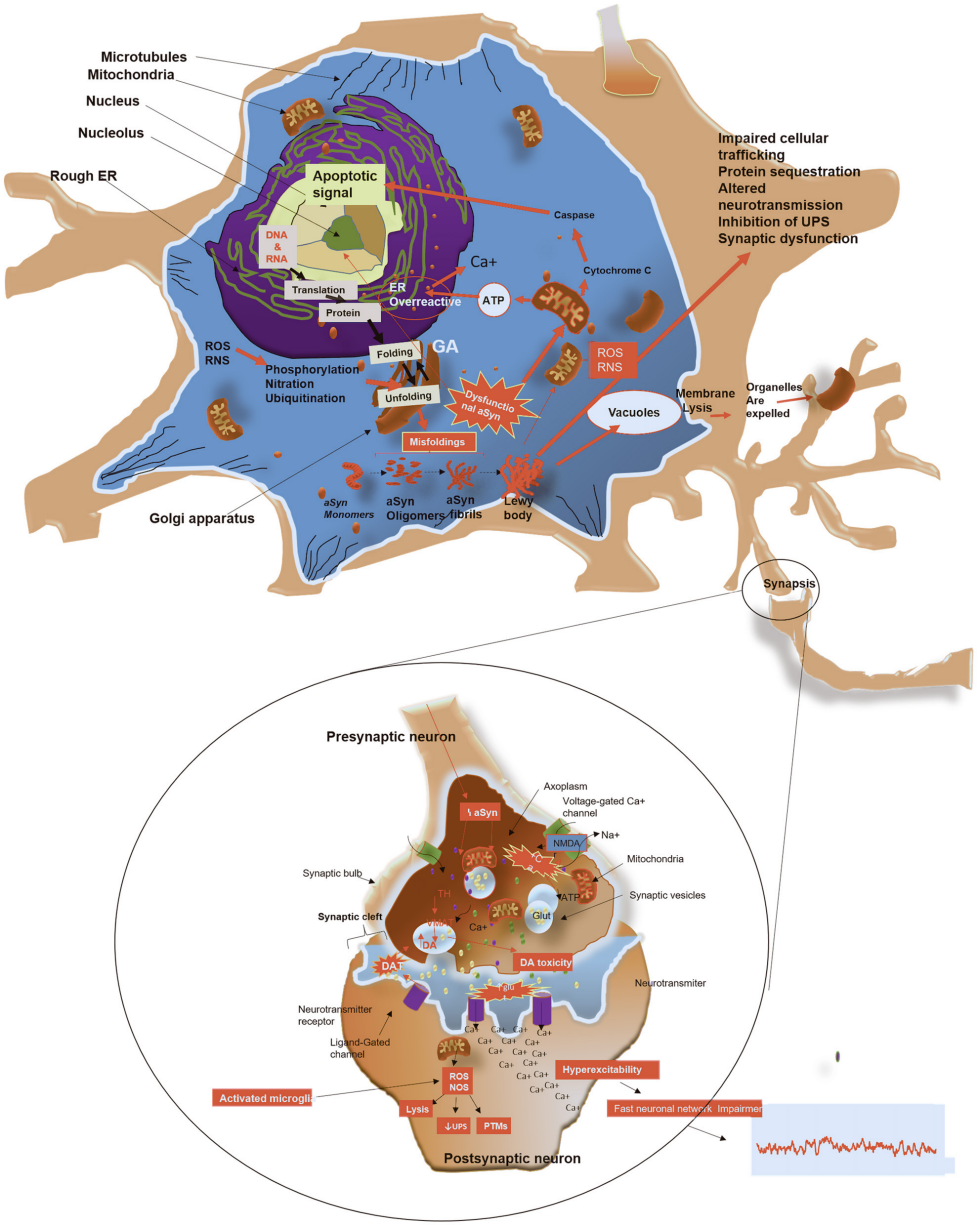
Schematic representation of potential mechanisms by which aSyn form aggregates, leads to toxicity and causes cell death. Monomeric aSyn assembles to form oligomers and producing mature fibrils. (1) LBs and LNs formation: fibrillary forms of aSyn are segregated into protein inclusions, which contain dysmorphic organelles, vesicular structures and various other cellular proteins, depleting cells of these vital components. (2) Mitochondrial impairment: Altered aSyn translocate to the mitochondria, causing oxidative stress by increasing production of reactive oxygen and nitrogen species with consequent impairment of energy production. Damage to mitochondria leads to release of Cytochrome C. This is in turn activates caspases, which translocate to the nucleus to initiate an apoptotic response. (3) ER dysfunction and protein trafficking inhibition: depletion of ATP due to mitochondrial dysfunction, causes ER overactivity leading to increased release of calcium. This further causes blockage of protein trafficking from ER to Golgi, leading to Golgi fragmentation. (4) Membrane pore formation: putative toxic forms of aSyn may penetrate cellular membranes, altering its permeability, causing excess of calcium and other ions within the cytosol. (5) Impaired autophagy: adhesion of aSyn to the membrane of lysosomes alters autophagy mediated by chaperones, resulting in aggregation of substrates and impairment of the proteasome system. (6) Release of toxic aSyn and other cellular components into extracellular space: toxic aSyn may be passively released in the extracellular space by dying neurons and consequently taken by adjacent neurons, resulting in seeding aggregation and synaptic impairments. (7) Defects in axonal transport: toxic aSyn causes Tau hyper phosphorylation, which inhibit the modulation of microtubule assembly, with consequent detrimental effects on cellular transport, increasing aggregation of toxic substances in the cytosol. (8) Synaptic terminal dysfunction: toxic aSyn alters distribution of proteins in the synaptic terminal, reduces synaptic vesicle release, which leads to changes of the synaptic protein composition and hyperexcitability. (9) Impairment in dopamine (DA) metabolism: aggregation of aSyn impairs metabolism of DA, which induces ROS. Figure was constructed using several references (Hampton, 2000; Lindersson et al., 2004; Danzer et al., 2007; Li W. et al., 2007; Prots et al., 2013; Shahmoradian et al., 2018).

Whilst it remains unclear whether the end-stage products of aggregation (LBs and LNs) are the most disease relevant toxic forms of aSyn, it is widely assumed that the process of aggregation is critical and perhaps its intermediates such as prefibrils or fibrillary oligomers formed during such a process are the toxic elements (Periquet et al., 2007). This is supported by the fact that, aSyn and β-synuclein fragments which do not contain the *NAC* domain, were found to be resistant to aggregation (Periquet et al., 2007). Moreover, aSyn oligomers and aggregated fibrils were found to exhibit toxicity even before forming LBs/LNs, and these aggregation precursors have been associated with progressive motor impairments and neuronal cell death (Periquet et al., 2007). Cell culture and *in vivo* studies in animal models have shown that aggregated fibrils cause further aggregation of aSyn monomers that consequently translocate between cells in prion-like manner (Wood et al., 1999; Desplats et al., 2009; Hansen et al., 2011). This finding indicates that aSyn pathogenic cascade, may begin even before LBs are formed, causing functional alterations before the structural changes are evident. Furthermore, aggregated aSyn was shown to affect various cellular pathways, impairing the adaptation of the neurons to endoplasmic reticulum (ER) stress, inducing dysfunction of the proteasome degradation process, mitochondria functioning, and leading to membrane damage as well as loss of synapses (Figure 3) (Wood et al., 1999; Desplats et al., 2009; Hansen et al., 2011).

### Dysfunction of the Ubiquitin-Dependent Proteasome Degradation System

It is expected that in the course of time, large quantities of misfolded aSyn aggregates, and accumulates within the neurons, consequently leading to inclusion formation. In physiological conditions as other proteins, aSyn should be eliminated before forming aggregates. This process normally occurs via different proteolytic pathways within cellular compartments, such as the lysosomal pathway or the ubiquitin dependent proteasome degradation system also known as the ubiquitin proteasome system (UPS), which is a major protein degradation pathway targeting misfolded and unwanted proteins (Hyttinen et al., 2014). UPS is mediated by a tri-enzymatic reaction that leads to the mono-ubiquitination or poly-ubiquitination of the target protein on lysine residues. Poly-ubiquitination is associated with protein degradation by the 29S proteasome and require substrate recognition by 19S proteasome subunit prior to degradation by the proteolytic core of the 20S proteasome complex (Tanaka and Chiba, 1998; Tanaka, 2009).

aSyn is found to be highly ubiquitinated in LBs (Lindersson et al., 2004). It has been indicated that, the accumulation of these proteins marks aSyn for degradation. Major components of the proteolytic 20S proteasome are widely present in LBs/LNs (Lindersson et al., 2004). Thus, it can be considered that these proteins have been sequestered from their physiological role to be degraded and/or eliminated. In addition or even preceding the incorporation of the 20S proteasomes into LBs/LNs, its degradation capacity may be impaired as result of the direct binding of aSyn oligomers (Nielsen et al., 2001; Tanaka, 2009; Emmanouilidou et al., 2010). This was proven in a study which showed that, aSyn oligomers prominently impair proteasomal activity in PC12 cells expressing mutant A53T (Zondler et al., 2017). Thus, supporting the notion that in pathologic cellular environments aSyn cannot be degraded by the proteasome system, and thus contributing to selective vulnerability of cells incorporating LBD pathology. The selective vulnerability of the substantia nigra to proteasome inhibition in PD patients, is thought to occur as a result of proteasomal inhibition by the oligomeric species of aSyn (McNaught and Jenner, 2001; McNaught et al., 2001; Olanow and McNaught, 2006). On the other hand, when proteasomal activity is enhanced, as it was demonstrated with a potent proteasome activator such as Tetramethylpyrazine Analog T-006, aSyn is efficiently degraded not only by the UPS but also in autophagy independent manner (Zhou et al., 2019). Other studies have demonstrated that the inhibition of UPS by aSyn was reversed with the supplementation of Congo red, which is an aSyn oligomerization inhibitor (Emmanouilidou et al., 2010). Thus, the selective vulnerability of aSyn to form aggregates is greatly enhanced by the failures of ubiquitin dependent clearance mechanisms.

### Mitochondrial Dysfunction and Membrane Damage

Although, aSyn is abundant in the cytosol, accumulating evidence suggests the presence of endogenous or overexpressed aSyn within mitochondria (Li W. W. et al., 2007; Nakamura et al., 2008; Liu et al., 2009; Loeb et al., 2010). This is thought to lead to aSyn related mitochondrial toxicity. Some studies proposed that aSyn binds to and inhibits the function of a subunit of the mitochondrial protein import system, such as TOM20 (Wiedemann et al., 2004; Wurm et al., 2011; Di Maio et al., 2016). On the other hand, mitochondrial damage can lead to oligomerization and aggregation of aSyn, thus revealing bidirectional toxicity (Betarbet et al., 2000; Cannon et al., 2009).

Moreover, aSyn shows high affinity with voltage-dependent anion channel (VDAC), which is an outer mitochondrial membrane channel that regulates flux of small hydrophilic molecules and calcium in and out of the mitochondria (Lu et al., 2013). Rostovtseva et al. (2015) showed that, aSyn monomers reversibly inhibit VDAC in a voltage dependent manner, creating a high influx of calcium into the mitochondria (Rostovtseva et al., 2015). The high influx of calcium into the mitochondria is thought to be facilitated by aSyn oligomers, which consequently propels mitochondrial swelling, that occurs as a result of depolarisation, and retention of exogenous calcium inside the mitochondrial matrix (Luth et al., 2014). Another study revealed that, overexpression and subsequent oligomerization of aSyn led to mitochondrial fragmentation in dopaminergic SH-SY5Y cell line (Plotegher et al., 2014). Mitochondrial fragmentation was also found to be induced by mutant forms of aSyn, and this is thought to be associated with excessive mitophagy within cells (Choubey et al., 2011; Nakamura et al., 2011). In addition, toxic aSyn species are thought to cause downregulation of complex I in the electron transport chain of mitochondria (Nakamura et al., 2008; Loeb et al., 2010). The effects of aSyn on mitochondria, particularly the inhibition of complex I, induces the production of reactive oxygen species (ROS) and oxidative stress which leads to the impairments of mitochondrial function, with consequent upregulation of apoptotic signals within the cell (Figure 3) (Hsu et al., 2000; Ryan et al., 2015). This suggests loss of mitochondria functions as a key part of a pathogenic process of LBDs.

Cell stability relies on the integrity of its membranes which function as a barrier between intracellular and extracellular environments, controlling the movement of the metabolites. When incubated with natural and synthetic phospholipids or other lipid bilayer membranes *in vitro*, aSyn rapidly formed aggregates and oligomeric species (Haque et al., 2010; Grey et al., 2011). In similitude to the mitochondria membrane, extracellular oligomeric aSyn species were shown to form pores on the cell membranes. This is thought to facilitate the influx of excessive exogenous calcium within the cytosol, which is a process that often precedes cellular disruption and death (Danzer et al., 2007). In addition, unfolded monomeric and oligomeric species of aSyn are thought to cause calcium dysregulation by interacting with the calcium membrane signaling in a site-specific manner (Angelova et al., 2016). Interestingly, only oligomeric species are capable of inducing cell death. It would be interesting to study if other pre-aggregated and/or aggregated forms have the same effects. Contrastingly, protecting the membrane with new compounds such as NPT100-18A, which can displace aSyn from the lipid bilayer membrane, reduces toxicity of oligomeric aSyn (Wrasidlo et al., 2016). Supporting this, endosulfine-alpha, a compound that selectively binds to membrane-associated aSyn, was found to block the formation of toxic aggregated forms within the cell and reduce the death of dopaminergic neurons in PD (Ysselstein et al., 2017). Thus, demonstrating that abnormal interaction between toxic aSyn with the membranes is linked with the impairment of the cellular membrane function in LBDs.

### aSyn and Synaptic Membrane Dysfunction

Synaptic dysfunction is an early event in the evolution of LBDs, as well as in other neurodegenerative diseases (Schulz-Schaeffer, 2010). Considering the physiological role of aSyn at the synapse, it is plausible that overexpressed and aggregated aSyn induces synaptic dysfunction and neurotoxicity. In contrast to physiological aSyn, which facilitates formation of the SNARE complex, and control movement of synaptic vesicles and release of neurotransmitters, oligomeric aSyn, acting via an association with synaptobrevin, inhibits SNARE complex formation. Thus preventing the fusion of synaptic vesicles with the plasma membranes and the subsequent release of neurotransmitters (Choi et al., 2013, 2018; Burré et al., 2018; Masaracchia et al., 2018; Man et al., 2021). Moreover, in COS-7 cells, aSyn overexpression was shown to cause disruption of the Golgi apparatus, the site of synaptic vesicle formation, and thus led to synaptic dysfunction by the depletion of synaptic vesicles (Gosavi et al., 2002). In addition, oligomeric species of aSyn were found to cause reduction in neurotransmitter release by inducing premature intracellular rupture of synaptic vesicles (Danzer et al., 2007). In a similar manner, aSyn oligomers promote increased calcium influx due to cell membrane permeabilization with consequent result of increased neuronal excitotoxicity (Danzer et al., 2007) (Figure 3). Furthermore, aSyn oligomers decreased the stability of microtubules (MT), which are fundamental for axonal transport and the regulation of neuronal homeostasis (Goldstein et al., 2008; Prots et al., 2013).

*In vitro* studies demonstrated that the interaction of aSyn with 3,4-dihydroxyphenylacetaldehyde (DOPA), a toxic product of dopamine degradation, promotes aSyn oligomerization (Burke, 2003). The consequence of this interaction is the permeabilization of cholesterol-rich lipid membranes, including synaptic vesicles (Burke, 2003). Altogether, these studies suggest a synergistic effect of DOPA formation and aSyn toxicity and may explain the selective vulnerability of dopaminergic neurons to the rupture of synaptic vesicles (Burke, 2003; Plotegher et al., 2017; Lima et al., 2019). Overall, the main effects of aggregated and oligomeric forms of aSyn are mediated by the reduction of neurotransmitter release, inhibition of synaptic vesicle recycling, enlargement of synaptic vesicle, loss of presynaptic proteins, and redistribution of the SNARE proteins (Chung et al., 2009; Garcia-Reitbock et al., 2010; Nemani et al., 2010). These are key pathogenic features that may underlie the synaptic impairment observed in LBDs.

### Endoplasmic Reticulum Stress and Membranes

Endoplasmic reticulum (ER) is the cellular site of protein synthesis, folding, modification, and release to the secretory pathway. Perturbation at any stage of ER-mediated proteostasis leads to ER stress (Hampton, 2000). ER stress is thought to occur early in the development of aSyn pathology. This hypothesis was tested in a study using A53T aSyn mutant expressing PC12 cell lines, which reported a lower effect of aSyn neurotoxicity in cells treated with Salubrinal, a pharmacological inhibitor of ER stress (Smith et al., 2005). In support of this, a study using SH-SY5Y cells showed that ER stress was linked with overexpressed aSyn (Castillo-Carranza et al., 2012). Oligomeric species of aSyn are also associated with increased levels of chronic ER stress transgenic mouse models of synucleinopathy (Colla et al., 2012; Colla, 2019). Moreover, Rab1, which is a protein that regulates the membrane trafficking, was found to be associated with cytoplasmic aSyn inclusions. Its role in aSyn-mediated neurotoxicity was demonstrated by a study using a yeast model of synucleinopathy by showed showing its synergic effects in blocking ER-Golgi vesicular trafficking (Cooper et al., 2006). Thus, the ER may be a primary target of aSyn toxicity.

**Figure 4:**
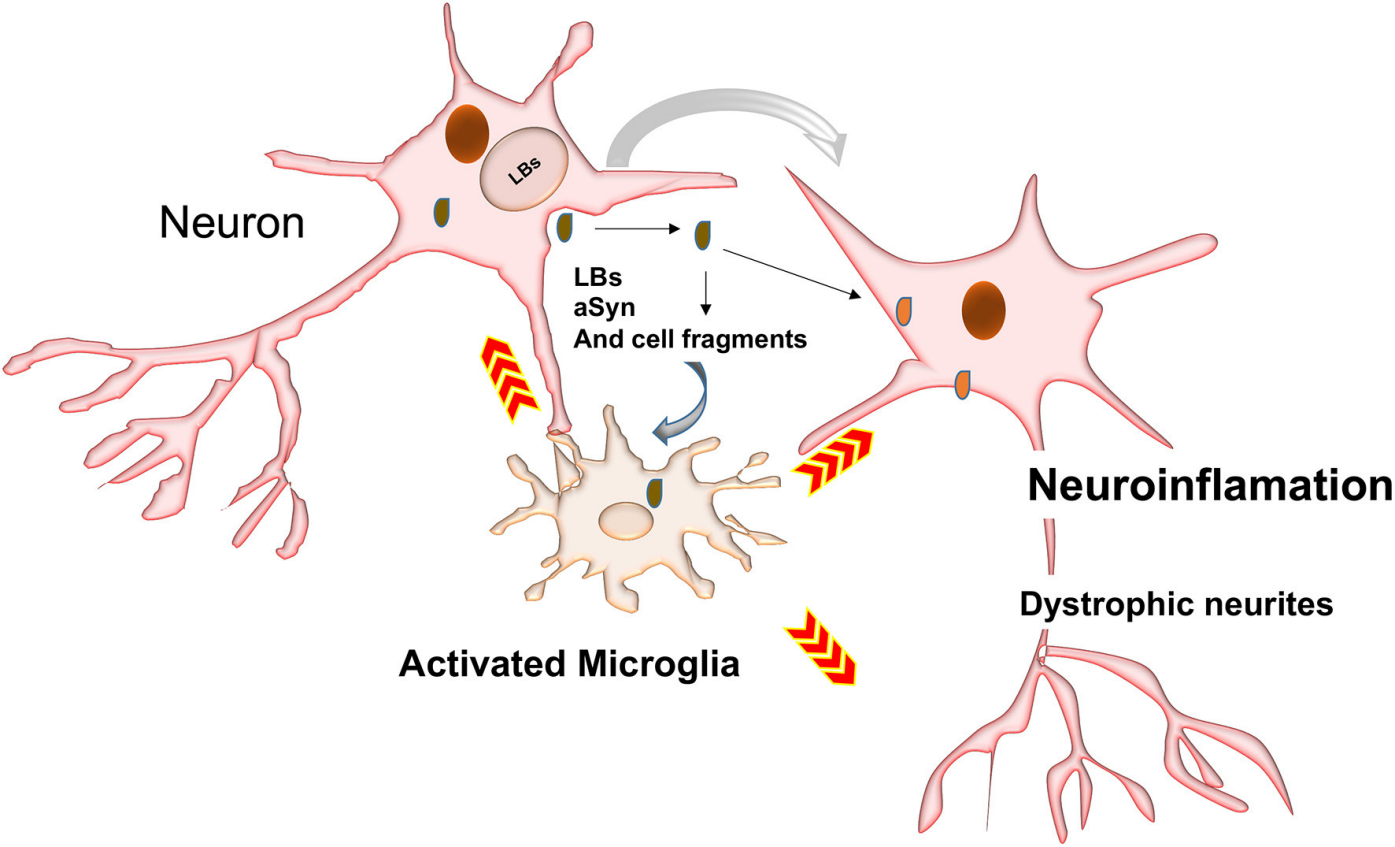
Possible mechanisms involved in activation of phagocytosis and inflammatory responses. LBs may be passively secreted in the extracellular environment following cell death by cell-to-cell transfer. Inclusions and their toxic intermediates may activate astrocytes and glia cells surrounding neurons, resulting in the release of pro-inflammatory cytokines and ROS, which could damage neighboring neurons.

Neurons may also release toxic aSyn species into the extracellular space (Qiao et al., 2012). These could be taken up by microglia (Figure 4) to prevent further ER stress and toxicity (Park et al., 2008). aSyn synergistically interacts with Toll-like receptor2 (TLR2), which is a potent microglia activator (Qiao et al., 2012; Kim et al., 2013; Stivers et al., 2017).

## Post-translational Modifications of aSyn

It is likely that loss of the dynamic equilibrium between the soluble, membrane-associated and aggregated forms of aSyn leads to aSyn toxicity and LBD pathology. Exactly how this phenomenon occurs, remains to be determined, and to address this, it is important to know first, what occurs before aSyn forms aggregates. We posit that the remarkable number of PTMs that is reported to occur in aSyn may underlie such disequilibrium. For a relatively small molecule, a large number of PTMs are reported to occur in the protein (Figure 2). PTMs are chemical modifications of amino acid residues that carry a potential to modify protein structure and regulate protein localization, activity, binding affinity, and degradation (Beck-Sickinger and Mörl, 2006; Prabakaran et al., 2012; Schmid et al., 2013). aSyn PTMs, such as ubiquitination, nitration (Oueslati et al., 2010), acetylation (Fauvet et al., 2012; Maltsev et al., 2012; Theillet et al., 2016), truncation (Li et al., 2005; Beyer and Ariza, 2013), SUMOylation (Dorval and Fraser, 2006; Wilkinson and Henley, 2010; Krumova et al., 2011), glutathionylation (Kim et al., 2012; Zhang et al., 2018), Glycosylation (Spiro, 2002), fatty acid acylation (Robinson et al., 1970; Resh, 2016), and phosphorylation, have been reported to occur in aSyn, and more will probably come in the future. To date, these PTMs have been studied in isolation and of all them, the most studied is aSyn phosphorylation at serine 129 (pSer^129^) (Oueslati et al., 2010). Nevertheless, the precise roles of these PTMs in LBD pathogenesis are the focus of much research, as this is still not well-understood (see Table 2).

**Table 2:** Known post-translational modifications in αSyn, sites, enzymes, and effects.

**Known PTMs**	**aSyn Residue**	**Enzymes**	**Cytotoxicity**	**References**
Phosphorylation	Y39, Y126, S129 Y133, Y136	CKII GRK2 PLK2 SIAH	Aggregation Oligomerization Degradation Aggregation	Okochi et al., 2000; Fujiwara et al., 2002; Arawaka et al., 2006; Waxman and Giasson, 2008; Oueslati et al., 2010; Paleologou et al., 2010; Mahul-Mellier et al., 2014
Ubiquitination	M1, K6, K10, K12, K21, K23 K32, K34, K43 K45, K96, K102	SIAH CHIP	Aggregation Degradation	Shin et al., 2005; Lee et al., 2008; Rott et al., 2008; Tetzlaff et al., 2008
Nitration	Y39, Y125, S129 Y133, Y136	–	Aggregation	Hodara et al., 2004
Truncation	K58, V74, K80 G84, A89, K97 E110, K102 P120, Y125	Calpain I Neurosin	Aggregation Polymerization Degradation	Mishizen-Eberz et al., 2005; Kasai et al., 2008
SUMOylation	K96 K102	PIAS2	Aggregation	Rott et al., 2017
o-GlcNAcylation	T33, T59, T64 T72, T75, T81 S87	OGT	Aggregation	Lewis et al., 2017; Levine et al., 2019
N-Acetylation	–	–	No difference in aggregation or membrane binding	Runfola et al., 2020

*For sites of aSyn residue refer to Figure 2. PIAS, protein inhibitor of activated STAT; CK II, casein kinase II; GRK2, G protein coupled receptor kinases; PLK2, polo like kinases 2; SIAH, seven in absentia homolog; CHIP, carboxyl terminus of Hsp 7-interacting; PIAS2, protein inhibitor of activated STAT 2; OGT, o-linked N-acetylglucosamine (GlcNAc) transferase*.

### aSyn Phosphorylation

Phosphorylation is a form of reversible PTM that plays an essential role in the regulation of physiological and pathological functions of proteins involved in pathways including but not limited to differentiation, cellular metabolism, gene expression, cell cycle progression, intercellular communication, cellular motility, migration, and cytoskeletal arrangements (Manning et al., 2002a,b). Under physiological conditions, the level of aSyn phosphorylation is very low, and the presence of phosphorylated residues such as tyrosine (pY^39, 125^) and serine (pSer^87^) are almost absent (Okochi et al., 2000; Fujiwara et al., 2002; Anderson et al., 2012). In contrast, phosphorylation of threonine (pT^125, 133, 135^), serine (pSer^87^, pSer^129^), and tyrosine (pY^125, 133, 136^) was detected in pathologically aggregated aSyn. Most of the phosphorylated residues are localized in *C terminus* of the protein, which is believed to be involved in aSyn pathology, except pSer^87^ (Chen et al., 2009, 2010; Venda et al., 2010; Xu et al., 2015). The most abundant phosphorylation PTM occurs at pSer^129^, which is now considered an important marker for synucleinopathies (Figure 2) (Okochi et al., 2000; Fujiwara et al., 2002; Takahashi et al., 2003; Anderson et al., 2006). In addition, this type of aSyn phosphorylation is found to be widespread within the LBs and LNs in corresponding regions of the brain affected in LBDs (Takahashi et al., 2003; Zhou et al., 2011; Walker et al., 2013).

aSyn phosphorylation is also linked to the formation of the toxic species of aSyn, leading to the rapid formation LBs (Kahle et al., 2000a,b; Okochi et al., 2000; Fujiwara et al., 2002; Anderson et al., 2006). In PD, over 90% of endogenous aSyn is found to be phosphorylated at Ser^129^; in direct contrast to only 4% aSyn phosphorylation detected in healthy individuals (Kahle et al., 2000b; Fujiwara et al., 2002). Similarly, pSer^129^ was found in post-mortem brain in LBDs subjects and in SH-SY5Y cells that overexpress wild-type aSyn (Swirski et al., 2014). A recent study demonstrated behavioral deficits associated to widespread pSer^129^ aSyn in brains of wild-type d transgenic mice that overexpress aSyn under Thy1-promoter (Gabrielyan et al., 2021), as pSer^129^ phospho-memetic reported slower/inhibited aggregation kinetic than wild-type aSyn, despite its prominence in LBs and the preferential phosphorylation of fibrillar aSyn. It has been, suggested that pSer^129^ is a late-stage event in aggregation leading to the fibrillization of aSyn, and pSer^129^ is deemed to be crucial for the regulation of disease progression (Paleologou et al., 2010; Waxman and Giasson, 2011; Wales et al., 2013). Other studies suggest different forms of phosphorylation upregulate each other. For instance, both pSer^87^ and pSer^129^ were found to be present in LBs aggregates and enhance aSyn toxic membrane interactions in synucleinopathies (Paleologou et al., 2010). Moreover, the binding of pSer^129^ with vesicular trafficking, cytoskeletal proteins and other phosphorylated linked enzymes, is thought to reinforce the important interrelationships among these pathways in the pathogenesis of LBDs, by interfering with functions of these proteins as well as utilizing such proteins as co-aggregates (McFarland et al., 2009). In addition, proteasomal inhibition (previously described to be a result of aSyn toxicity), was reported to increase the toxicity of aSyn pSer^129^, as well as its toxicity in dopaminergic neurons in PD. This suggests there is more rapid degradation mechanism of phosphorylated aSyn under physiological conditions (Chau et al., 2009; Oueslati et al., 2013; Mahul-Mellier et al., 2014; Arawaka et al., 2017). Hence, it can be surmised that phosphorylation of aSyn, particularly at residue 129, may be a key factor in the formation of LBs and the development of LBD.

#### Kinases Involved in aSyn Phosphorylation

In all cellular environments, including the extracellular matrix, phosphorylation process is enzymatically balanced by protein kinases and phosphatases (Bononi et al., 2011; Datta and Ganapathy, 2017). Several kinases are known to be involved in aSyn Ser^129^ phosphorylation, including casein kinases (CK1, CK2) (Okochi et al., 2000; Ishii et al., 2007; Takahashi et al., 2007; Waxman and Giasson, 2008, 2011), polo like kinase 2 (PLK2, also known as serum inducible kinase (SNK) (Inglis et al., 2009), and G protein-coupled receptor kinases (GRK1, GRK2, GRK5, and GRK6) (Pronin et al., 2000; Arawaka et al., 2006; Sakamoto et al., 2009). The co-localization of GRK5 and aSyn was observed in LBs/LNs inclusions in post-mortem brains of PD patients (Arawaka et al., 2006). However, this was not corroborated in DLB (Arawaka et al., 2006). Moreover, GRK5 knockdown in SH-SY5Y cells failed to completely suppress aSyn phosphorylation (Liu et al., 2010). On the other hand, CK1 and CK2 were found to induce aSyn phosphorylation in mammalian cells, in a yeast model and in rat primary cortical neurons (Okochi et al., 2000; Ishii et al., 2007; Waxman and Giasson, 2008; Zabrocki et al., 2008). Furthermore, oxidative stress increases aSyn phosphorylation mediated by CK2 and its autologues, resulting in an intensification in the formation of LBs/LNs inclusions in SH-SY5Y cells (Pronin et al., 2000). Similarly, PLKs, a family of kinases known to be essential for cellular response to carcinogenesis, stress response, and cell cycle regulation (Ng et al., 2006), were found to participate in aSyn Pser^129^ (Inglis et al., 2009; Mbefo et al., 2010). Although yet to be confirmed by others, Leucine-rich repeat kinase 2 (LRRK2), an enzyme involved in the pathogenesis of PD, was reported to directly interact with and phosphorylate aSyn (Table 2) (Qing et al., 2009).

#### aSyn Phosphorylation and Oxidative Stress

Increasing evidence indicates an association between aSyn aggregation and oxidative stress. It has been suggested that the latter has a direct causal relationship with aSyn phosphorylation (Chau et al., 2009). In SH-SY5Y cell line, treatment with low doses of rotenone, an electron transport chain complex I inhibitor that causes an increase in reactive oxygen species (ROS) production, led to increased levels of aSyn pSer^129^ (Sugeno et al., 2008). Similarly, an *in vitro* study of a synucleinopathy model demonstrated that aSyn phosphorylation increases when exposed to rotenone and ferrous iron (Perfeito et al., 2014). The increase in aSyn phosphorylation was also observed in SH-SY5Y cells exposed to exogenous toxins such as 6-hydroxidopamine, epoxomicin (a proteasome inhibitor), and paraquat (an environmental toxin that causes oxidative stress) (Chau et al., 2009; Ganapathy et al., 2016). Perfeito et al. (2014), hypothesized that stimuli that promote mitochondrial dysfunction and the formation of ROS are associated with mutant A53T aSyn pSer^129^. They found correlation between rotenone and aSyn pSer^129^ in the formation of reactive oxygen species which may underlie neuronal degeneration in LBDs (Perfeito et al., 2014). Thus, biochemical changes linked with LBDs pathogenesis such as oxidative stress, mitochondrial complex I dysfunction, and proteasome impairments, could underlie changes in aSyn phosphorylation (Lee and Trojanowski, 2006; Lashuel et al., 2012; Robson et al., 2018).

#### Subcellular Distribution of Phosphorylated aSyn

aSyn is a predominantly cytosolic protein but is also found in subcellular organelles such as mitochondria. Whether aSyn localizes within the nucleus is still under debate. Notwithstanding, studies performed in rat and mouse models of synucleinopathy showed that phosphorylated aSyn localized in the nucleus of DA neurons in hydroxydopamine treated rats (Yamada et al., 2004; Wakamatsu et al., 2007). Moreover, pSer129 was shown to modulate the interchange of aSyn between the nucleus and cytoplasm in human neuroglioma cells (Outeiro et al., 2008). This is supported the finding that the translocation dynamics of aSyn between the cytosol and the nucleus was altered upon co-expression of aSyn with various kinases (reviewed in Goncalves and Outeiro, 2013). Correspondingly, cytoplasmic and nuclear shuttling of aSyn was also found to be modulated by GRK5, PLK2, and PLK3 (Table 2) (Monti et al., 2010). In addition, the phospho-mimicking Ser^129^A and Ser^129^D were found to localize in the nucleus and increase cellular toxicity (Ganapathy et al., 2016). Pathological insults such as oxidative stress that induce increased aSyn phosphorylation have shown to promote nuclear aSyn localization (Monti et al., 2010; Siddiqui et al., 2012; Ganapathy et al., 2016). Although, we still do not know the exact function of aSyn within the nucleus, it is believed that once aSyn translocates to the nucleus, it acts as a transcriptional regulator via interacting with PGC-1alpha and histones, thus enhancing the toxicity of aSyn in the nucleus (Goers et al., 2003; Kontopoulos et al., 2006; Vasquez et al., 2017; Schaser et al., 2019).

#### Phosphorylated aSyn as Biomarker in Synucleinopathies

The efficiency of phosphorylated aSyn as a potential biomarker is being widely explored in the diagnosis of LBDs. Quantification of total aSyn in the cerebrospinal fluid (CSF) in patients diagnosed with synucleinopathies and related disorders was proposed as a potential biomarker particularly in differentiating early stages of other related pathologies (Mollenhauer et al., 2008; Hong et al., 2010). However, recent study reported that in LBDs, aSyn pSer^129^ was found present in the human plasma but not in the CSF (Cariulo et al., 2019). Nevertheless, according to recent discoveries phosphorylated aSyn, improves the performance of aSyn as biomarker on other tissues. For instance, aSyn pSer^129^ in the retina was proposed as a biomarker for PD, as it was found to accumulate in parallel with aSyn pSer^129^ in the brain (Ortuño-Lizarán et al., 2018). Moreover, skin biopsies performed in PD subjects, has proven to be a reliable tool for the detection of aSyn pSer^129^ to discriminate LBDs from controls (Tang et al., 2020). Another important diagnostic test that could also be used for LBDs is the detection of phosphorylated aSyn in the peripheral nervous system, which revealed aggregation of phosphorylated aSyn in large and small fibers (Wang et al., 2012; Doppler et al., 2014; Stewart et al., 2015). Furthermore, aggregation of phosphorylated aSyn was detected in the gastrointestinal tract of PD subjects (Pouclet et al., 2012; Hilton et al., 2014). Although, Foulds et al. (2013), showed that the total number of aSyn in the blood plasma of PD patients were similar to the findings in non-PD controls. The levels of aSyn pSer^129^ were remarkably higher in PD patients compared to non-PD controls (Foulds et al., 2013; Cariulo et al., 2019). A more recent study has reported aSyn pSer^129^ in red blood cells, could be used as a potential biomarker, as it was found to be higher in patients with synucleinopathy (Xu et al., 2015; Cariulo et al., 2019; Li et al., 2020). Thus, phosphsphorylated aSyn is perhaps a more applicable candidate for the development of biomarkers for the diagnosis of LBDs.

### aSyn Ubiquitination

Intraneuronal compartments are highly specialized to facilitate many operations such as cell cycle, transcription, transduction, antigen presentation, protein degradation, and apoptosis (Hershko and Ciechanover, 1998). Many of these processes require ubiquitin, which is a small protein with 76 amino acid residue that is ubiquitously expressed in the nervous system (Varshavsky, 2001). Ubiquitination, is a PTMs that regulates protein abundance inside the neurons, in a process by which ubiquitin is covalently conjugated to a substrate protein, in the presence of ubiquitin activating enzyme (E1-UBA), ubiquitin conjugating enzyme (E2-UBC) and a ubiquitin ligase (E3-UBL) (Stone, 2016). The process terminates with the formation of an amide bond between ubiquitin and lysine or in other instances with cysteine, serine or threonine residues of the substrate protein (Wang et al., 2007; Vosper et al., 2009). When the conjugation culminates with the single molecule of ubiquitin attached to the residue protein, the process is referred to as mono-ubiquitination. If the process is repeated, the addition of several ubiquitin molecules leads to the formation of a poly-ubiquitin chain. Poly-ubiquitination leads to protein degradation, primarily by the UPS, and it sometimes uses macro-autophagy as an alternative pathway linked to degradation of long-lived ubiquitinated proteins, protein aggregates and organelles (Wang and Pickart, 2005; Hochstrasser, 2006; Li W. et al., 2007; Deshaies and Joazeiro, 2009; Maspero et al., 2011).

Ubiquitinated aSyn is often present in the LBs and LNs inclusions. In fact, multiple lines of evidence have reported the presence of mono-ubiquitinated aSyn as a predominant species in LBs and LNs (Lowe et al., 1990; Wakabayashi et al., 2000; Hasegawa et al., 2002; Tofaris et al., 2003; Anderson et al., 2006). Although, mono-ubiquitination mechanisms that underlie the modulation of aSyn aggregation are still unknown, a study on isolated LBs and LNs from PD patients detected the presence of the seven in absentia homolog (SIAH), an E3 ubiquitin ligase enzyme, previously reported to interact with and ubiquitinated aSyn (Liani et al., 2004; Engelender, 2008; Lee et al., 2008). De facto, lysine 12, 21, and 23 residues, the common targets of aSyn mono ubiquitination, are also ubiquitinated by SIAH (Anderson et al., 2006). This suggests SIAH is involved in the ubiquitination of aSyn, contributing to LBD pathology. However, this remains to be confirmed because some studies have found that only a small fraction of aSyn is ubiquitinated in LBs and LNs (Hasegawa et al., 2002).

In LBDs, neurons are heavily laden with proteasome subunits and ubiquitinated structures, suggesting their involvement and impairments in their degradation (Kuzuhara et al., 1988; Bentea et al., 2017; Lehtonen et al., 2019). Although UPS and the autophagy-lysosomal pathways constitute two major degradation pathways which manage the elimination of misfolded, aggregated, and damaged neuronal proteins (Seglen et al., 1996; Ciechanover and Kwon, 2015; Bustamante et al., 2018; Blasiak et al., 2019), the UPS, chaperone-mediated autophagy (CMA), and macroautophagy all appear to be involved in the proteolytic degradation of aSyn. CMA is proposed to be the main mechanism involved in aSyn clearance (Bennett et al., 1999; Paxinou et al., 2001; Webb et al., 2003).

### aSyn SUMOylation

aSyn SUMOylation occurs when a lysine residue of aSyn is covalently linked with a small ubiquitin-like modifier (SUMO) protein. SUMO proteins share structural and mechanistic similarity with ubiquitin (Jentsch and Pyrowolakis, 2000; Dohmen, 2004). Like ubiquitin, SUMO forms covalent links with its substrates through isopeptide bonds between glycine and lysine residues, using similar enzymes to those that mediate the conjugation of ubiquitin (Jentsch and Pyrowolakis, 2000; Dohmen, 2004). This process is believed to be associated with the pathological formation of proteinaceous inclusions within neurons (Dohmen, 2004). In fact, abnormal aSyn SUMOylation was detected in many neurodegenerative diseases such as LBDs, Huntington's disease, AD, amyotrophic lateral sclerosis (Figure 4) (Fei et al., 2006; Kim et al., 2011; Krumova et al., 2011; O'Rourke et al., 2013; Luo et al., 2014; Ochaba et al., 2016). aSyn is conjugated to SUMO at lysine residues (K6, K10, K12, K21, K23, K34, K45, K60, K80, K96, and K102) (Dorval and Fraser, 2006; Kim et al., 2011; Krumova et al., 2011; Coelho-Silva et al., 2017; Iyer and Claessens, 2019). Two studies have demonstrated that aSyn SUMOylation is a signal for its proteasome-mediated degradation and proteasome inhibition led to aggregation of SUMOylated aSyn (Table 2) (Kim et al., 2011; Oh et al., 2011). Additionally, aSyn SUMOylation levels were increased in LBs/LNs in LBD cases (Rott et al., 2017).

It has been demonstrated that E3 SUMO-protein ligase Protein inhibitor of activated STATS (PIAS2) SUMOylates aSyn, thus promoting aSyn aggregation, and an increase in the levels of extracellular aSyn, preceding the formation of the proteinaceous inclusions (Kunadt et al., 2015). It is believed that the increase of extracellular aSyn is a consequence of SUMOylation build up in the endosomal complex, which is linked with aSyn efflux (Kunadt et al., 2015). This process was also demonstrated to occur in the absence of proteasome inhibitors (Kunadt et al., 2015; Rott et al., 2017). SUMOylation is thought to impair the process of mono/poly ubiquitination of aSyn mediated by SIAH and Nedd4 (Liani et al., 2004; Rott et al., 2008, 2017). Two ligases found to be involved in aSyn are SUMOylation, E3 SUMO-protein ligase PIAS, and E3 SUMO-protein ligase PIAS2, as their increase was markedly detected in PD brains (Rott et al., 2017). It is thought that PIAS2 promotes SUMOylation of aSyn, thus inhibiting the action of SIAH and Nedd4 ubiquitin ligases, leading to a decrease of aSyn Ubiquitination, causing aSyn aggregation and LBs formation (Rott et al., 2017). Therefore, it is suggested that these enzymes upregulate aSyn SUMOylation, which promotes overexpression of the protein, thus increasing its aggregation and toxicity.

### aSyn Nitration

Protein nitration is a nitrosative stress related PTM that affects the tyrosine (Tyr) residues (Radi, 2004; Bartesaghi and Radi, 2018). aSyn is nitrated at its C terminal in residues Y^125^, Y^133^, Y^136^, and at N terminal residue Y^39^ (Figure 2) (Giasson et al., 2000; Souza et al., 2000). These Tyrosine residues are found to be necessary for aSyn aggregation, especially under oxidative stress conditions (Souza et al., 2000). Indeed, it is believed that aSyn exposure to nitrating agents leads to the formation of covalently stable oligomers through the oxidation of tyrosine to o,o'-dityrosine (Souza et al., 2000). Although, high rates of oligomerization were observed upon Tyr^39^ nitration, studies have shown that Tyr^125^ is more relevant to aSyn dimer formation (Takahashi et al., 2002; Olteanu and Pielak, 2004; Yu et al., 2004; Danielson et al., 2011). Moreover, fibril formation is increased by both monomeric and dimeric-nitrated aSyn (Hodara et al., 2004).

### aSyn Nitration-Mediated Interaction With Membranes and Oxidative Stress

The association of aSyn with membranes is thought to lead to peroxidation of polyunsaturated fatty acids, as membrane lipid peroxidation can be triggered by ROS and RNOS. aSyn incubation with 4-hydroxy-2-nonenal (4HNE), a lipid peroxidation marker, leads to the formation of aSyn-HNE adducts (Trostchansky et al., 2006; Qin et al., 2007). Some studies have suggested that the interaction of aSyn with membrane phospholipids and polyunsaturated fatty acids is necessary for the formation of aSyn oligomers (Perrin et al., 2001; Assayag et al., 2007; Beyer and Ariza, 2013).

aSyn also promotes nitric oxides (NO) activity at the plasma membrane, which leads to NOS production. This excess of NOS is thought to inhibit the electron transport chain, thus increasing the production of superoxide (Lashuel et al., 2002). This creates a vicious cycle that facilitates nitration and oxidation that potentiate cellular dysfunction and loss of viability (Radi et al., 2002; Radi, 2004). Likewise, aSyn oxidation and nitration impedes the autophagy-mediated degradation, therefore inhibiting the degradation of nitrated aSyn (Martinez-Vicente et al., 2008). This resistance to degradation of nitrated aSyn promotes its intracellular increase in half life and consequently its concentration, which boosts the toxicity of the protein, stimulating neuronal degeneration (Chavarria and Souza, 2013).

### aSyn o-GlcNAcylation

O-beta-N-acetyl-D-glucosaminyl transferase (O-GlcNAc/ OGT) is an enzyme that catalyzes the glycosylation of the serine and threonine aa residues in a dynamic that is opposed by O-GlcNAcase (OGA) (Hart et al., 2007). O-GlcNAc of aSyn was detected on multiple threonine residues (33, 34, 54, 59, 64, 72, 75, 81, and 87) of aSyn extracted from human tissues of individuals with LBD and mouse models of synucleinopathies (Wang et al., 2009, 2010, 2017; Alfaro et al., 2012; Morris et al., 2015). Although, glycation exerts a large inhibitory effect on unmodified aSyn, it was, however, found to promote aggregation of pathological aSyn and increase the toxicity of the protein (Levine et al., 2019). Moreover, *in vivo* and *in vitro* studies performed in *Drosophila* and in A53T mouse model have shown that glycation potentiates the toxicity of aSyn by affecting its *N-terminal* domain, impairing the clearance of aSyn. This is thought to decrease the membrane binding properties of aSyn and enhancing the aggregation of its oligomeric species, altogether resulting in the impairment of synaptic transmission (Table 2) (Vicente Miranda et al., 2017). In DLB, aSyn o-GlcNAcylation was found to induce the formation and aggregation of its oligomeric forms (Zhang et al., 2017). Conversely, other studies revealed that, use of glycation inhibitors decreased aSyn aggregation, restored aSyn clearance, and abated clinical phenotype of the disease (Vicente Miranda et al., 2017).

### aSyn Truncation

Truncated forms of aSyn are ubiquitous in LBs inclusions. This is thought to be associated with abnormal proteolysis of aSyn at the *C terminus* of the protein (Tofaris et al., 2003, 2006; Periquet et al., 2007). aSyn C-terminal truncation is thought to occur through the lysosomal-associated pathways (Murray et al., 2003; Sevlever et al., 2008; Tsujimura et al., 2015; Sorrentino et al., 2018). Nevertheless, the mechanism by which truncated aSyn contributes to the formation of LBs and LNs inclusions remains to be elucidated. However, it is believed, that the amount of truncated aSyn species represents about 15% of all aSyn in LBs/LNs inclusions, and they may act as seeds that enhance the aggregation of aSyn to form the LBs/LNs under physiological pH (van der Wateren et al., 2018; Zhang et al., 2019). *In vivo* studies using transgenic mice and *Drosophila* that overexpress αSyn, also demonstrated that truncated aSyn is associated with increased aggregation of aSyn and neurotoxicity (Tofaris et al., 2003, 2006; Periquet et al., 2007). Interestingly, truncated aSyn was found to be important for the prion-like propagation property of toxic aSyn, which is believed to be at the root of the phenotypic diversity of the synucleinopathies (Terada et al., 2018). Indeed, in brains of DLB subjects the full-length as well as truncated and insoluble aggregates of aSyn are evident in LBs and LNs (Baba et al., 1998).

Furthermore, enzymes such as calpin1, cathepsin D, matrix metalloproteinase 3, and neurosin are involved in aSyn truncation (Table 2) (Iwata et al., 2003; Mishizen-Eberz et al., 2003; Dufty et al., 2007; Sevlever et al., 2008; Choi et al., 2011; Weber et al., 2019). aSyn is thought to be a substrate of calpain 1, a neural calcium-activated protease. Calpain 1 cleaves wild-type aSyn at amino acids 57 and 73, 74, and 83 located within the *NAC region*. This process promotes aggregation, therefore calpain 1 is thought to generate aggregation-prone forms of aSyn promoting fibril formation (Mishizen-Eberz et al., 2003). Similarly, neurosin, a protease that is ubiquitously expressed in brain cells and cleaves aSyn after aa 80 also increases the propensity of aSyn to form aggregates (Iwata et al., 2003; Kasai et al., 2008). These observations strongly support the involvement of truncated aSyn in the formation LBs/LNs and selective neuronal degeneration seen in DLB.

## Conclusions

aSyn aggregation and transformation into LBs and LNs underlie the onset and progression of LBDs and related synucleinopathies. It represents a failure in proteostasis network directly linked to maintain biologically active aSyn and reduce toxic forms of the protein. Multiple lines of evidence suggest PTMs are key in aSyn aggregation and toxicity across the synucleinopathies. The most studied PTMs associated with the LBDs are phosphorylation, ubiquitination SUMOylation, Nitration, o-GlcNacylation, and truncation. Under normal physiological conditions PTMs are known regulators of protein biology which is important for localization and function. However, recent findings demonstrate that they are associated with toxic species of aSyn with the consequent formation of LBs and LNs, disrupting cellular homeostasis leading to neuronal cell death.

Further understanding of the exact role of PTMs in LBDs is warranted. To date, few reports have largely focused on PD. Most studies reported thus far, are concentrated on non-acetylated aSyn, which needs more validation. It is therefore difficult to assess the full modulation of PTMs with the existing models of PTMs. Studies on specific PTMs using purified forms of aSyn are also essential, as this would permit more detailed study of each PTM to uncover the mechanisms of how PTMs modify aggregation and form LBDs. It is still unclear what role aSyn PTMs play in the propagation of LBD pathology. Nonetheless, the reports so far of the biological relevance of aSyn PTMs, has highlighted their importance in the pathogenesis of disease. This could lead to development of interventions aimed at reducing the accumulation of LBs and slowing or preventing LBDs. PTMs represent a more selective potential therapeutic target that addresses LB accumulation and therefore more specific for DLB, rather than simply be duplicative of AD or PD directed treatments.

## Author Contributions

NM: established concept, searched references (~300 articles from PubMed, PNAS databases, selecting 270), curation of data, writing original draft, and editing several version. LS: reviewing and editing drafts. RK: reviewing references and editing several drafts of the article. All authors contributed to the article and approved the submitted version.

## Conflict of Interest

The authors declare that the research was conducted in the absence of any commercial or financial relationships that could be construed as a potential conflict of interest.

## Abbreviations

DLB: Dementia with Lewy Bodies
LBDs: Lewy body disorders
LBs: Lewy bodies
LNs: Lewy neurites
PD: Parkinson's disease
PDD: Parkinson's disease dementia
ETC: electron transport chain
PTMs: post-translational modifications
aSyn: Alpha-synuclein
AA: amino acid
CSP: α-Cysteine Stirring Protein alpha
SNARE: Soluble NSF attachment receptor
UPS: Ubiquitin proteasome system
VDAC: voltage-dependent anion channel
ROS: reactive oxygen species
RNOS: reactive nitrogen species
MT: Microtubules
ER: Endoplasmic reticulum
TLR2: Toll-like receptor2.
